# Ten recommendations for creating usable bioinformatics command line software

**DOI:** 10.1186/2047-217X-2-15

**Published:** 2013-11-13

**Authors:** Torsten Seemann

**Affiliations:** 1 Victorian Bioinformatics Consortium, Building 76 Monash University Clayton, Clayton, VIC 3800, Australia; 2 Life Sciences Computation Centre, Victorian Life Sciences Computation Initiative, 187 Grattan St, Carlton, Melbourne, VIC 3010, Australia

**Keywords:** Bioinformatics software, Software quality, User interface, Unix, Tools

## Abstract

Bioinformatics software varies greatly in quality. In terms of usability, the command line interface is the first experience a user will have of a tool. Unfortunately, this is often also the last time a tool will be used. Here I present ten recommendations for command line software author’s tools to follow, which I believe would greatly improve the uptake and usability of their products, waste less user’s time, and improve the quality of scientific analyses.

## Background

New bioinformatics tools are released and published every day, most of which are designed for the Unix command line. Ignoring the important issue of algorithmic correctness, the first barrier for community uptake of a bioinformatics tool is the command line interface and usability. It is this author’s experience that the majority of these tools fail basic requirements of usability; and thus, a course of action to overcome this would be a list of minimum standards for all command line scientific software that would help software authors and reviewers to improve the average usability of released software tools.

I have used and installed a *lot* of bioinformatics software over the last 12 years, and I have also released a lot of my own software - I try to make it as painless to use as possible. From these experiences, I present ten recommendations for bioinformatics software, using the fictitious “BioTool” project as an example.

### *1. Print something if no parameters are supplied*

Unless your tool acts as a filter or pipe by manipulating *stdin* to *stdout*, you should always print some help text if the user runs your tool without the required parameters. Do not just exit silently.

### *2. Always have a “-h” or “--help” switch*

The Unix tradition is for all commands to have a *-h* or *--help* switch which prints usage information about the command [1]. Most programming languages provide a POSIX compliant *getopt()* library [2], so there is no excuse for not supporting this.

### *3. Have a “-v” or “--version” switch*

Many bioinformatics tools today are used as part of larger pipelines, or placed into the Galaxy Tool Shed [3]. The ability to reproduce an analysis depends critically on the specific version of a tool. There should be a simple, machine-parsable way to determine the version of a tool you have. If the tool resides in a public revision control system like GitHub [4] the branch hash can be used as a version string.

### *4. Do not use stdout for messages and errors*

If you need to print an error message, are just printing out progress or log information, use *stderr* rather than *stdout*. Please reserve *stdout* for your output channel, so that it can be used in Unix pipes (where possible) to avoid temporary files.

### *5. Always raise an error if something goes wrong*

When a Unix process finishes, it returns an integer exit code. Standard practice is to use zero for success, and non-zero for failure [5]. All tools should exit with a semantically correct exit code, so that the pipelines, shell scripts or Makefiles, they are embedded within, can detect when they fail and act accordingly.

### *6. Validate your parameters*

If you have command line options, do some validation or sanity checking on them before letting them through to your critical code. Many *getopt()* libraries support basic validation, but even a simple *if/then/exit* block is sufficient to catch invalid parameters.

### *7. Don’t hard-code any paths*

Often the tool you write depends on some other files, such as configuration or database files. The easiest, but wrong and annoying, thing to do is hard-code the absolute path in your source code.

A better solution is to locate your dependent files relative to where the main tool is installed. This can be done manually, or via a helper module like Perl’s FindBin [6].

### *8. Don’t pollute the PATH*

Often *biotool* is just a master script which runs other subtools in your BioTool package. Unfortunately if the subtools use generic names and they are in the same folder as *biotool*, the PATH is polluted unnecessarily. Please do not do this.

There are various solutions to this problem:

1. Use only one master command, which is used to invoke sub-commands. This is used effectively by popular software like *SAMtools*[7].

2. Prefix all your sub-tools and helper scripts with the name *biotool-*.

3. Ensure internal helper scripts are non-executable, so they don’t get indexed in the PATH, and instead invoke the scripts explicitly from *biotool*.

4. Place them in a separate sub-folder (eg., auxiliary/, scripts/) and explicitly call them (but take note of rule #7 above).

### *9. Check that your dependencies are installed*

It is frustrating to install BioTool, and start using it seemingly successfully, only to find it many hours later spit out an error like “*error: failed to run bwa*”. This is avoidable if *biotool* checks it has access to all the external tools it needs before it commences. This will save your users associating your software with frustration and wasted time.

### *10. Don’t distribute bare JAR files*

If your command line Java tool is distributed as a JAR file, please write a simple shell wrapper script to make it simple to invoke. The two lines below are all you need (in the simple case) and you will make your users much happier. It also enables you to specify sensible memory defaults too.

## Conclusion

Implementation of these recommendations would greatly improve the usability of command line bioinformatics software, and otherwise excellent ideas and tools would get the audience they deserve, rather than be ignored in frustration.

## Abbreviations

JAR: Java archive.

## Competing interests

The author declares he has no competing interests.

## Authors’ contributions

TS was the sole contributor to this article. The author has read and approved the final manuscript.

## Authors’ information

TS received a PhD in computer science in 2002, and joined the Victorian Bioinformatics Consortium as a junior scientist. He has worked in the field of microbial genomics and bioinformatics for over 10 years, and has written many open-source software tools for the analysis of genomics data. He is currently the Scientific Director of the Victorian Bioinformatics Consortium, and a senior researcher at the Life Sciences Computation Centre in Melbourne, Australia.
